# Research on the application of cerebral blood flow reconstruction technology in the surgical treatment of moyamoya disease

**DOI:** 10.3389/fsurg.2026.1726401

**Published:** 2026-01-26

**Authors:** Yuyang Chen, Chaojue Huang, Song Wu, Chang Liu, Yu Luo, Litian Huang, Guan Cao, Hui Liang, Panlin Mo, Jiachao Lu, Xiangsheng Su, Xiaoguang Tong, Daqin Feng, Tang Li

**Affiliations:** 1 Department of Neurosurgery, Wuming Hospital Affiliated to Guangxi Medical University, Nanning, China; 2 Department of Neurosurgery, The First Affiliated Hospital, Guangxi Medical University, Nanning, China; 3 Department of Neurosurgery, Tianjin Huanhu Hospital, Tianjin, China

**Keywords:** combined blood flow reconstruction, middle cerebral artery, moyamoya disease, superficial temporal artery, temporoparietal fascia flap

## Abstract

**Purpose:**

Moyamoya disease (MMD) is characterized by stenosis and occlusion of the cerebral arteries, leading to chronic progressive narrowing at the termini of the bilateral internal carotid arteries (ICA). It represents one of the primary etiologies for both ischemic and hemorrhagic strokes ( 1, 2).Currently, the primary treatment strategy for moyamoya disease (MMD) remains blood flow reconstruction surgery utilizing a bypass from the superficial temporal artery (STA) to the middle cerebral artery (MCA). This approach encompasses direct blood flow reconstruction, indirect blood flow reconstruction, and combined methods of blood flow reconstruction ( 3). However, there remains a paucity of high-quality results from clinical randomized controlled trials. This study aims to summarize the key points, selection criteria, and therapeutic effects of various surgical methods, thereby providing a foundation and accumulating experience for moyamoya disease (MMD) surgery.

**Method:**

A retrospective analysis was conducted on the clinical data of adult patients with moyamoya disease who underwent superficial temporal artery-middle cerebral artery + brain-muscle fusion (EMS) combined with blood flow reconstruction (EMS group) and superficial temporal artery-middle cerebral artery + pedicled temporoparietal fascia flap (TPFF) combined with blood flow reconstruction (TPFF group). Summarize and analyze the differences in technical characteristics, clinical efficacy, complications and prognosis between the two surgical methods; A retrospective analysis was conducted on the clinical data of adult patients with moyamoya disease who underwent the double-vessel ST-MCA + EMS group (double-vessel group) and the single-vessel ST-MCA + EMS group (single-vessel group). The technical characteristics, clinical efficacy, complications and prognosis differences of the two surgical methods were summarized and analyzed.

**Result:**

The incidence of postoperative epilepsy in the TPFF group was significantly lower than that in the EMS group (*P* = 0.043). There were no statistically significant differences between the two groups in terms of postoperative complications, mRS Scores, postoperative bypass patency rate, and Matsushima classification during postoperative follow-up (*P* > 0.05). There were no significant differences in mRS Scores, anastomotic patency rate of bypass vessels after surgery, CTP perfusion imaging indicators, and Matsushima between the double-vessel group and the single-vessel group (*P* > 0.05).

**Conclusion:**

This study further confirmed that STA-MCA + TPFF is a safe and effective combined blood flow reconstruction surgical method for the treatment of adult MMD, and it has the advantages of less trauma, simple operation, and avoiding adverse reactions caused by temporal muscle application, and can be used for clinical promotion and application. Both double and single STA-MCA + EMS can effectively improve cerebral perfusion in adult MMD patients. Individualized and reasonable choices should be made based on specific conditions.

## 1 Introduction

Extracranial and intracranial blood ow reconstruction represents the primary treatment approach for Moyamoya disease and Moyamoya syndrome (1, 2). The surgical techniques employed in blood flow reconstruction encompass direct blood flow reconstruction, indirect blood flow reconstruction, and combined surgical methods. Direct blood flow reconstruction entails a direct bypass between vessels, whereas indirect blood flow reconstruction does not involve direct vascular anastomosis. Instead, it facilitates the formation of new vessels by attaching pedunculated grafts to the surface of the pia mater of the brain, thereby contributing to intracranial blood supply and ameliorating the original ischemic condition. Direct blood flow reconstruction enhances perfusion within the distribution area of the middle cerebral artery (3). Indirect blood flow reconstruction procedures include: Encephalo-duro-arterio-synangiosis (EDAS), encephalo-myo-synangiosis (EMS), encephalomyo-arterio-synangiosis (EMAS), encephalo-duro-arterio-myo-synangiosis a(EDAMS), encephalo-duro-myo-synangiosis (EDMS), multiple-burr-hole (MBH), omental transposition (OT), with temporoparietal fascial flap (TPFF), etc. (4). Combined surgery refers to the simultaneous implementation of direct and indirect vascular reconstruction surgeries.

The use of the temporoparietal fascia flap (TPFF) for vascular reconstruction in moyamoya disease has been rarely reported in the literature (4). Upon searching databases such as PubMed with the keywords “TPFF” and “moyamoya disease,” we found that some international scholars have documented the application of TPFF in indirect and combined revascularization surgeries for moyamoya disease, achieving certain clinical outcomes. However, to date, there have been no clinical research reports on TPFF and moyamoya disease within China. This technique combines a large area of coverage for indirect revascularization with the benefits of a direct bypass. The pedicled TPFF also benefits from intact venous drainage to minimize the risk of flap swelling that could result in complications from mass effect (5). Although the process of indirect blood flow reconstruction is gradual, it possesses significant potential for enhancing blood supply, particularly in pediatric patients who exhibit a heightened capacity for angiogenesis. There is limited literature addressing the application of pedicled temporoparietal fascia flap combined with blood flow reconstruction surgery in the treatment of Moyamoya disease. This surgical approach offers several advantages: (1) The procedure can be performed without the need for additional incisions or injuries. The superficial temporal artery is located within the temporoparietal fascia, eliminating the necessity to separately dissect this artery. This approach not only conserves time but also simplifies the acquisition process. (2) The temporoparietal fascia is thinner than the temporal muscle, which eliminates the risk of space-occupying effects due to postoperative edema of the temporal muscle or postoperative epilepsy induced by electromyography. (3) The temporoparietal fascia is characterized by a rich vascular composition, which includes the superficial temporal artery, the superficial temporal vein, and an extensive capillary network. This anatomical structure exhibits significant potential for angiogenesis and possesses a buffering capacity that facilitates the automatic regulation of blood flow. Such properties may contribute to a reduction in postoperative complications, including hyperperfusion syndrome and bleeding. (4) There were no complications such as decreased aesthetic appearance or chewing function resulting from temporal muscle defects following the operation. However, potential drawbacks include complications like local alopecia due to hair follicle damage and poor incision healing, although these occurrences are relatively infrequent. Therefore, it is essential to perform dissection under a high-power microscope to minimize hair follicle injury and ensure that the flap maintains adequate thickness. During the procedure, care must be taken to preserve the pedicle at approximately 1 cm in width to avoid damaging the superficial temporal vein that runs alongside the superficial temporal artery. When dissecting along the anterior edge of the flap, it should closely adhere to the anterior margin of the frontal branch of the superficial temporal artery in order to prevent injury to this branch of the facial nerve. To mitigate postoperative hyperperfusion, a single anastomosis of the superficial temporal artery is typically sufficient in most cases.

Currently, there is a limited number of studies comparing the therapeutic effects of two surgical methods: STA-MCA combined with EMS and STA-MCA combined with TPFF. This study has confirmed that the combination of STA-MCA and TPFF represents a safe and effective approach for blood flow reconstruction in adult MMD patients. This method offers several advantages, including reduced trauma, simplicity in operation, and avoidance of adverse reactions associated with temporal muscle application, making it suitable for clinical promotion. Furthermore, both double or single STA-MCA combined with EMS have been shown to significantly enhance cerebral perfusion in adult MMD patients. Therefore, individualized selection should be made based on the specific conditions presented by each patient.

Thorough preoperative assessment of patients with Moyamoya disease, along with the individualized selection of appropriate surgical methods based on each patient's condition, and effective perioperative management are critically important for minimizing perioperative complications and enhancing the clinical prognosis of these patients.

## 2 Objects and methods

### 2.1 Object

This study retrospectively analyzed the medical records from two clinical research centers: From January 2018 to June 2022, a total of 79 MMD patients who were diagnosed (6) and treated with STA-MCA + EMS in the Department of Neurosurgery of the First Affiliated Hospital of Guangxi Medical University and those who were diagnosed and treated with STA-MCA + TPFF in the Department of Neurosurgery of Tianjin Huanhu Hospital during the same period were admitted. This study complies with the Declaration of Helsinki and was approved by the Ethics Committee of the First Affiliated Hospital of Guangxi Medical University and the Ethics Committee of Tianjin Huanhu Hospital respectively. All patients included in the study and their legally designated guardians signed written informed consent forms. Classify these clinical data, conduct postoperative follow-up, analyze the relevant clinical data results, and retrospectively summarize and analyze the surgical efficacy and impact of different surgical methods on MMD patients.

### 2.2 Data collection and grouping of research subjects

All the patients included in the study were aged 18 years or older, had no history of drug or alcohol abuse, and underwent systematic assessment, including medical history, neurological examination, laboratory tests, and imaging examinations (DSA, CTA, CTP). From gender, age, previous basic medical history (history of hypertension, diabetes, hyperlipidemia), classification of initial clinical manifestations (ischemic type, hemorrhagic type), specific clinical manifestations (including limb weakness, aphasia, blurred vision, dizziness, etc.), Suzuki stage, CTP perfusion indicators (CBF, MTT), surgical methods of the patients, postoperative complications, mRS Scores before surgery, 1 week after surgery and 6 months after surgery (preoperative and postoperative neurological function assessment of the patients) were statistically analyzed. The included cases were divided into the STA-MCA combined with EMS surgery group (hereinafter referred to as the “EMS group”) and the STA-MCA combined with TPFF surgery group (hereinafter referred to as the “TPFF group”).The clinical data and postoperative follow-up results of the two combined blood flow reconstruction surgical methods in the EMS group and the TPFF group were compared and analyzed to explore the advantages, disadvantages and clinical application value of the two combined blood flow reconstruction surgical methods. The patients in the EMS group were further divided into the single-vessel STA-MCA bypass combined with EMS application group (hereinafter referred to as the “single-vessel group”) and the double-vessel STA-MCA bypass combined with EMS application group (hereinafter referred to as the “double-vessel group”). The safety, clinical efficacy and prognosis of STA-MCA bypass in the single-vessel and double-vessel groups for the treatment of MMD were analyzed. The surgical methods and fluorescence angiography methods are described in the Supplementary Materials.

### 2.3 Postoperative assessment and follow-up methods

On the first day after the operation, a head CT scan was reexamined. Combined with clinical manifestations, the patient was evaluated for perioperative complications such as cerebral infarction, cerebral hemorrhage, epilepsy, and poor healing of the surgical incision after the operation. Within one week after the operation, patients with MMD underwent head and neck CTA or DSA and cranial CTP perfusion imaging to evaluate the patency of the STA-MCA anastomosis, collateral circulation compensation, and cerebral perfusion status in the surgical area (CBF, MTT), respectively. The CBF and MTT in the area around the anastomosis of the MCA on the surgical side were measured in the cranial CTP perfusion imaging. The modified Rankin Scale score was evaluated at discharge (mRS). Outpatient or telephone follow-up was conducted 6 months after the operation to assess mRS And whether there were any new complications (such as cerebral infarction, cerebral hemorrhage, epilepsy, etc.); The first imaging follow-up was completed 6 months after the operation. Cranial MRI plain scan, CT perfusion imaging (CTP), and digital subtraction angiography (DSA) were reexamined to evaluate whether new cerebral infarction occurred, the patency rate of bypass vessels, and the Matsushima grade of DSA. The CBF and MTT in the area around the anastomosis of the surgical side MCA in cranial CTP perfusion imaging were measured.

### 2.4 Data statistics and analysis methods

All the data in this study were statistically analyzed using statistical software SPSS (IBM SPSS Statistics for Windows, Version 25.0) and Prism9. Measurement data were expressed as mean ± standard deviation (mean ± SD). For the comparison between the two groups, the independent sample t-test is used for the data that conform to the normal distribution. The categorical variable is represented as N (%). Chi-square test or Fisher's exact test was used for comparison between groups, and Mann–Whitney *U* test was used for comparison between independent groups and non-normally distributed dat. To compare the CBF and MTT values of the single-vessel bypass group and the double-vessel bypass group at different time points, one-way repeated measures (AMOVA) analysis of variance was used. When *P* < 0.05, it is considered statistically significant.

## 3 Result

### 3.1 Analysis of clinical data of the STA-MCA + EMS group and the STA-MCA + TPFF group

**Figure 1:**
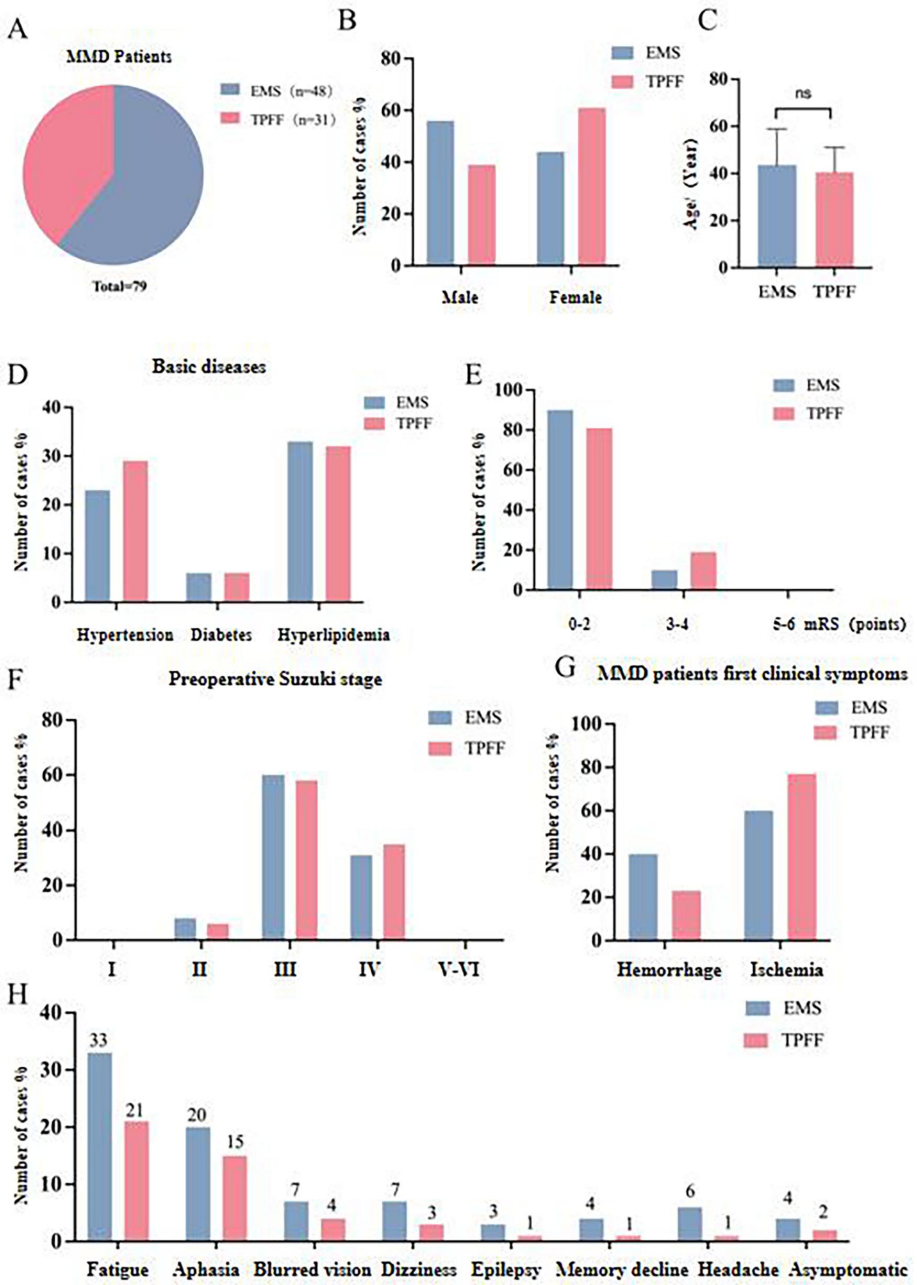
General clinical data statistics of patients in the STA-MCA + EMS group and those who underwent STA-MCA + TPFF application **(A)** the proportion of cases in each group of the EMS group and the TPFF group **(B)** comparison of gender between the two groups; **(C)** the age range of each group; **(D)** the previous underlying medical history of MMD patients; **(E)** the proportion of patients with different preoperative mRS score levels in the EMS group and the TPFF group; **(F)** the proportion of patients with different Suzuki stages before surgery in the EMS group and the TPFF group; **(G)** the initial clinical symptoms of MMD patients in the EMS group and the TPFF group; **(H)** the distribution of preoperative clinical manifestations in the EMS group and the TPFF group.

A total of 79 patients with MMD moyamoya disease were included in this study and divided into the STA-MCA + EMS group (referred to as the “EMS group”) and the STA-MCA + TPFF application group (referred to as the “TPFF group” (Supplementary Table S1, Figure 1A), Among them, there were 48 cases in the EMS group and 31 cases in the TPFF group. In the EMS group, there were 27 males and 31 females. There were 12 male patients and 19 female patients in the TPFF group (Supplementary Table S1, Figure 1B) (*P* = 0.196).The median age of the patients included in the study was 42 (29.75, 51) years in the EMS group and 39 (30, 52) years in the TPFF group (*P* = 0.98) (Supplementary Table S1, Figure 1C). The initial clinical symptoms of the patients were classified into hemorrhagic and ischemic types. In the EMS group, 19 cases (40%) presented with hemorrhagic type, 29 cases (60%) presented with hemorrhagic type, 7 cases (23%) presented with hemorrhagic type in TPFF, and 24 cases (77%) presented with hemorrhagic type (*P* = 0.185) (Supplementary Table S1, Figure 1G). The specific clinical manifestations of the included patients included fatigue, aphasia, blurred vision, dizziness, epilepsy, memory decline, headache, asymptomatic symptoms, etc. (Supplementary Table S1, Figure 1H). The mRS Scores of the two groups of patients were mainly 0–2 points. There were 43 cases (90%) in the EMS group and 25 cases (81%) in the TPFF group. Among the patients with mRS Scores of 3–4 points, there were 5 cases (10%) in the EMS group and 6 cases (19%) in the TPFF group (Figure 1F) (*P* = 0.325).The Suzuki stages of the included patients were mainly concentrated in stages II to IV. In the EMS group, 4 cases (8%) presented as stage II, 29 cases (60%) as stage III, and 15 cases (30%) as stage IV. In the TPFF group, 2 cases (6%) presented as stage II and 18 cases (58%) as stage III. There were 11 cases (35%) in stage IV (*P* = 0.937) (Figure 1F).There were no significant differences between the two groups of patients in terms of surgical side, gender, age, mRS Score, initial clinical symptoms, clinical manifestations, Suzuki stage, etc. (*P* > 0.05) (Supplementary Table S1, Figure 1).

### 3.2 Comparative analysis of postoperative efficacy and follow-up results between the STA-MCA + EMS group and the STA-MCA + TPFF group

**Figure 2:**
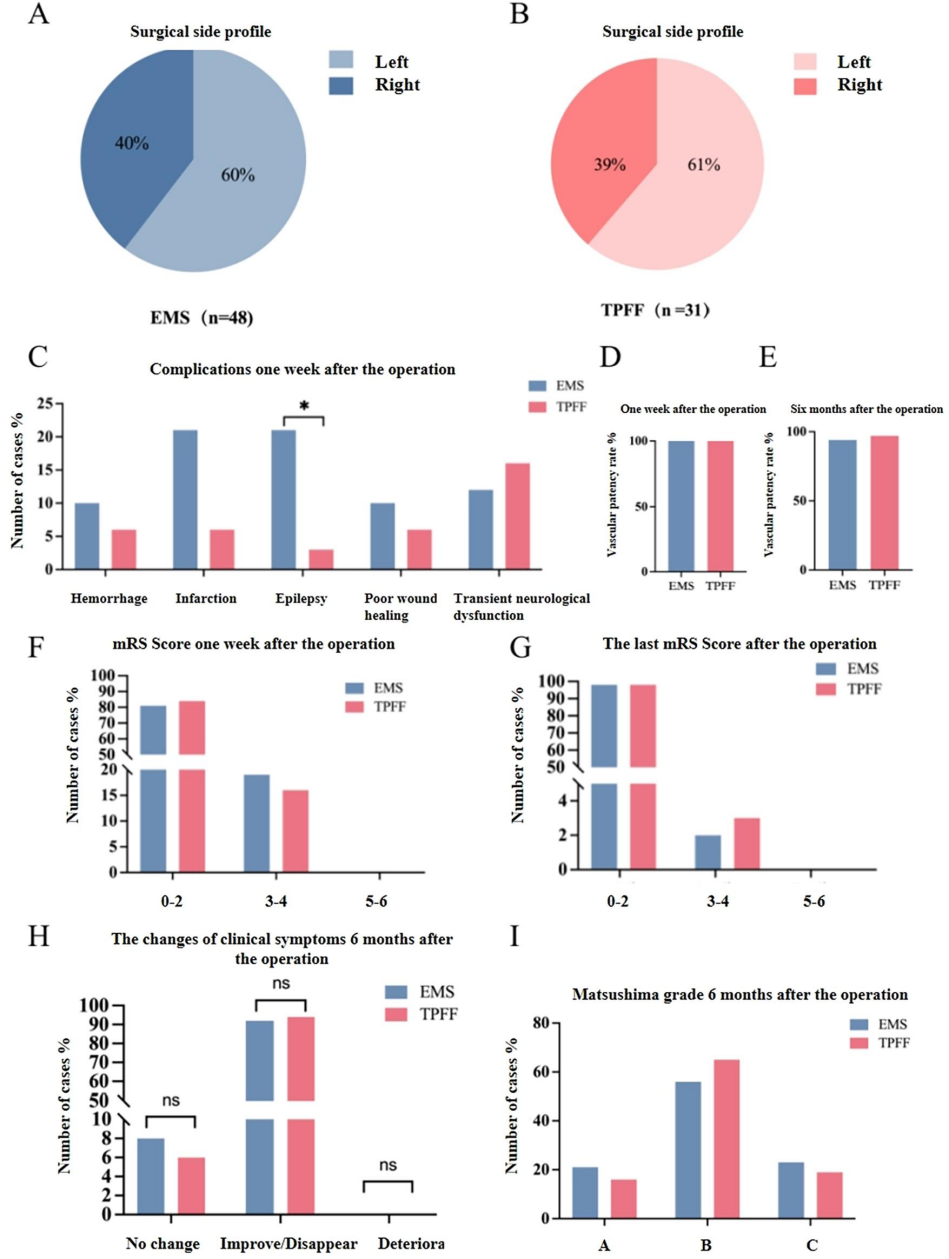
Analysis of postoperative follow-up results of patients in the STA-MCA + EMS group and the STA-MCA + TPFF group **(A)** the proportion of cases on the surgical side in the EMS group; **(B)** the proportion of cases on the surgical side in the TPFF group; **(C)** the proportion of complications that occurred one week after surgery in the EMS group and the TPFF group; **(D)** the patency rate of bypass vascular anastomosis one week after surgery in the EMS group and the TPFF group; **(E)** the patency rate of bypass vascular anastomosis one week after surgery in the EMS group and the TPFF group; **(F)** the mRS scores of the EMS group and the TPFF group one week after the operation; **(G)** mRS scores of the EMS group and the TPFF group at 6 months after surgery; **(H)** the improvement degrees of various clinical symptoms after surgery in the EMS group and the TPFF group; **(I)** the Matsushima classification of the EMS group and the TPFF group at 6 months after surgery.

Further analysis of the relevant follow-up data of patients in the EMS group and the TPFF group after surgery (Supplementary Table S2), In the EMS group, the lesion locations of 19 patients (40%) were on the left side (dominant side), while those of 29 patients (60%) were on the right side (Figure 2A); The number of patients with lesions located on the left and right sides in the TPFF group was 19 (61%) and 12 (39%), respectively (Figure 2B). One week after the operation, the probability of epilepsy in the TPFF group was significantly lower than that in the EMS group (*P* = 0.043).However, there were no significant differences in cerebral hemorrhage (*P* = 0.698), cerebral infarction (*P* = 0.112), poor incision healing (*P* = 0.698), and transient neurological dysfunction (*P* = 0.744) (Supplementary Table S2, Figure 2C). In terms of clinical symptom improvement (Supplementary Table S2, Figure 2F), including no change in symptoms (8% in the EMS group and 6% in the TPFF group), improve/disappear (92% in the EMS group and 94% in the TPFF group), and deteriora (0 in both the EMS group and the TPFF group), there was no difference between the two groups (*P* = 1). At the same time point, there was no significant difference in mRS Scores between the EMS group and the TPFF group (Figures 2F,G). In the long-term follow-up, DSA showed that the anastomosis rate of the bypass vessels in the EMS group one week after surgery was 100%, but it slightly decreased at 6 months after surgery, to 94% in the EMS group and 97% in the TPFF group (Figures 2D,E). Six months after the operation, there were 10 cases (21%) of patients with Matsushima grade A in the EMS group and 5 cases (16%) in the TPFF group; Among the patients of grade B, there were 27 cases (56%) in the EMS group and 20 cases (65%) in the TPFF group, The number of patients classified as grade C in the two groups was 11 cases (23%) and 6 cases (19%) respectively (*P* = 0.761).

### 3.3 Comparative analysis of the postoperative efficacy and follow-up results of double-vessel and single-vessel STA-MCA bypass combined with EMS

**Figure 3:**
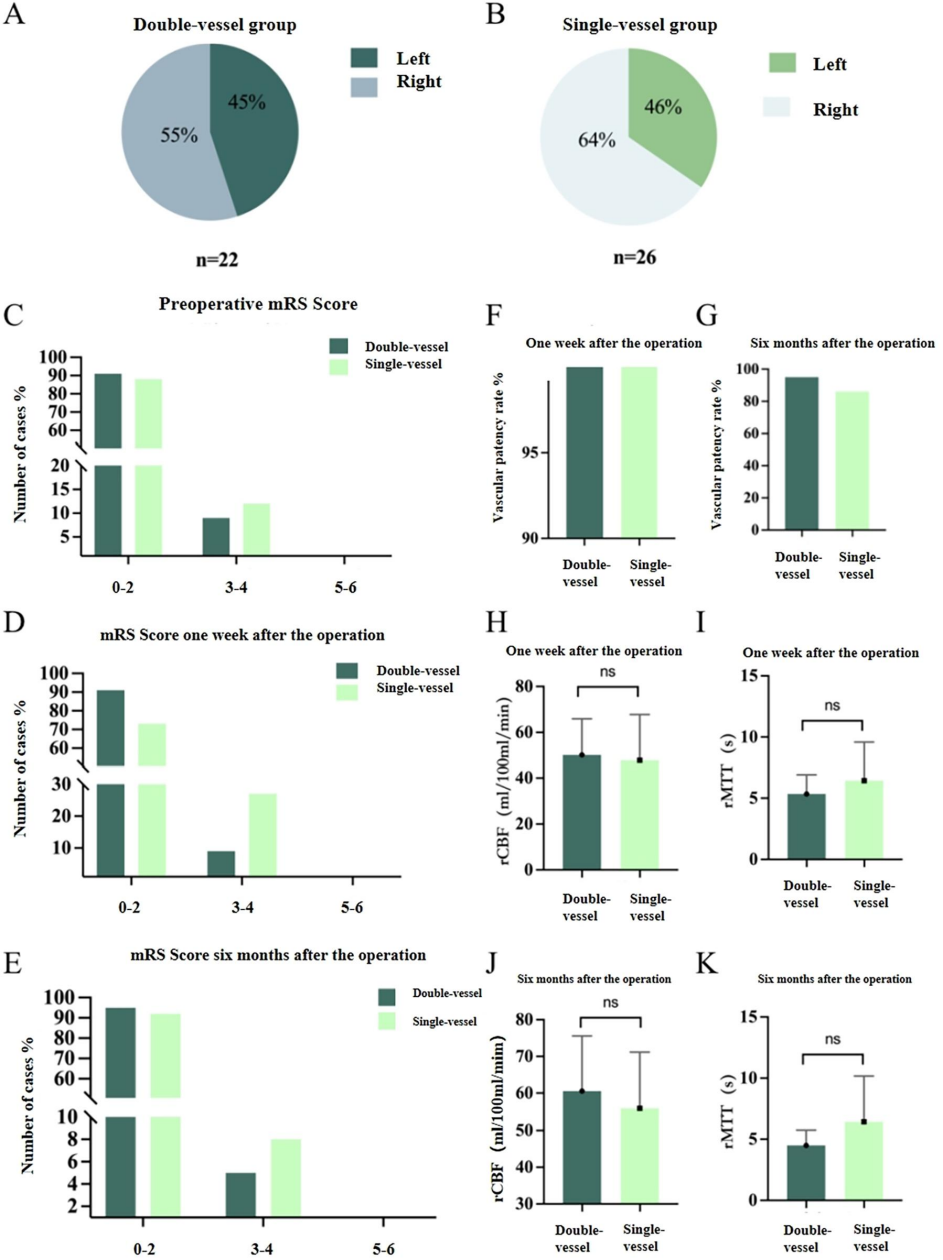
Follow-up analysis of the double-vessel STA-MCA bypass group and the single-vessel STA-MCA bypass **(A)** the proportion of surgical sides in the double-vessel group; **(B)** the proportion of surgical sides in the single-vessel group; **(C)** the mRS scores before and after surgery in the double-vessel group and the single-vessel group; **(D)** the mRS scores of the double-vessel group and the single-vessel group one week after the operation; **(E)** the mRS scores of the double-vessel group and the single-vessel group at 6 months after surgery; **(F)** the patency rate of bypass vascular anastomosis one week after surgery in the double-vessel group and the single-vessel group; **(G)** the patency rate of bypass vascular anastomosis 6 months after surgery in the double-vessel group and the single-vessel group; **(H)** the CBF changes in the double-vessel group and the single-vessel group one week after the operation; **(I)** the MTT changes in the double-vessel group and the single-vessel group one week after the operation; **(J)** the CBF changes in the double-vessel group and the single-vessel group one week after the operation; **(K)** MTT changes in the double-vessel group and the single-vessel group at 6 months after surgery.

**Figure 4:**
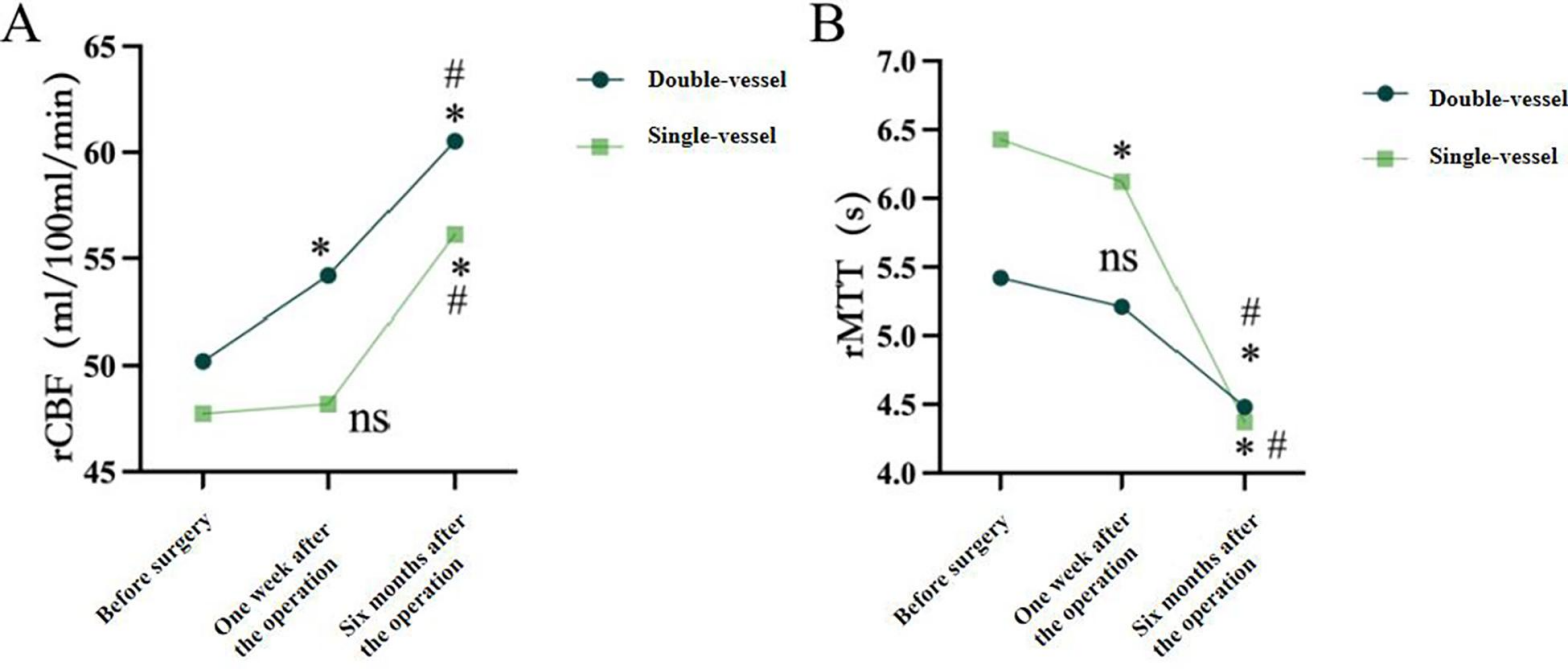
Comparison of CTP indicators of blood perfusion changes after double-vessel STA-MCA bypass surgery and single-vessel STA-MCA bypass surgery **(A)** the CBF changes of the double-vessel group and the single-vessel group before the operation, 1 week after the operation, and 6 months after the operation; **(B)** changes in the MTT assessment of the double-vessel group and the single-vessel group before the operation, 1 week after the operation, and 6 months after the operation. Data are expressed as mean ± standard deviation. Compared with that before the operation, * indicates *P* < 0.05; Compared with 1 week after the operation, # indicates *P* < 0.05 (The double-stent STA-MCA bypass group is denoted as the “double-vessel group”, and the single-stent STA-MCA bypass group is denoted as the “single-vessel group”).

**Figure 5:**
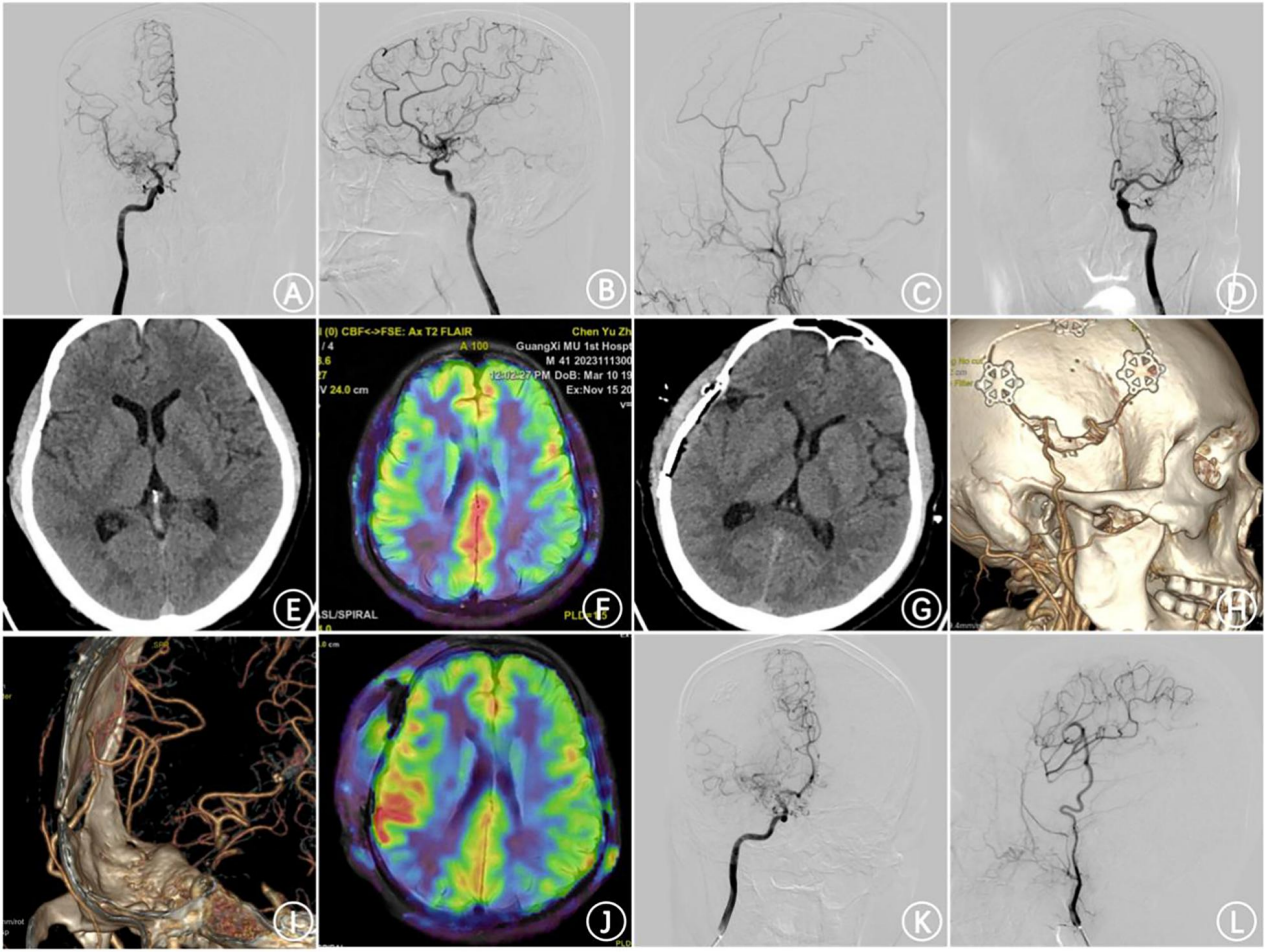
Imaging follow-up of cases of STA-MCA + EMS combined blood flow reconstruction surgery **(A,B)** preoperative DSA indicated stenosis at the terminal end of the right internal carotid artery and smoke vessel formation; **(C)** preoperative DSA lateral angiography of the left external carotid artery showed no obvious compensation; **(D)** preoperative DSA indicated that no obvious abnormalities were found in the left internal carotid artery angiography; **(E)** preoperative CT plain scan showed no obvious abnormalities; **(F)** preoperative perfusion imaging indicated a decrease in CBF in the right hemisphere; **(G)** no obvious abnormalities were found in the CT re-examination on the first day after the operation; **(H,I)** one week after the operation, the reexamination of CTA indicated that the STA-MCA anastomosis was unobstructed; **(J)** one week after the operation, perfusion imaging indicated that the CBF in the right hemisphere was higher than that before the operation; **(K,L)** the DSA reexamination half a year after the operation indicated that the STA was significantly thickened and supplied blood to the MCA.

**Figure 6:**
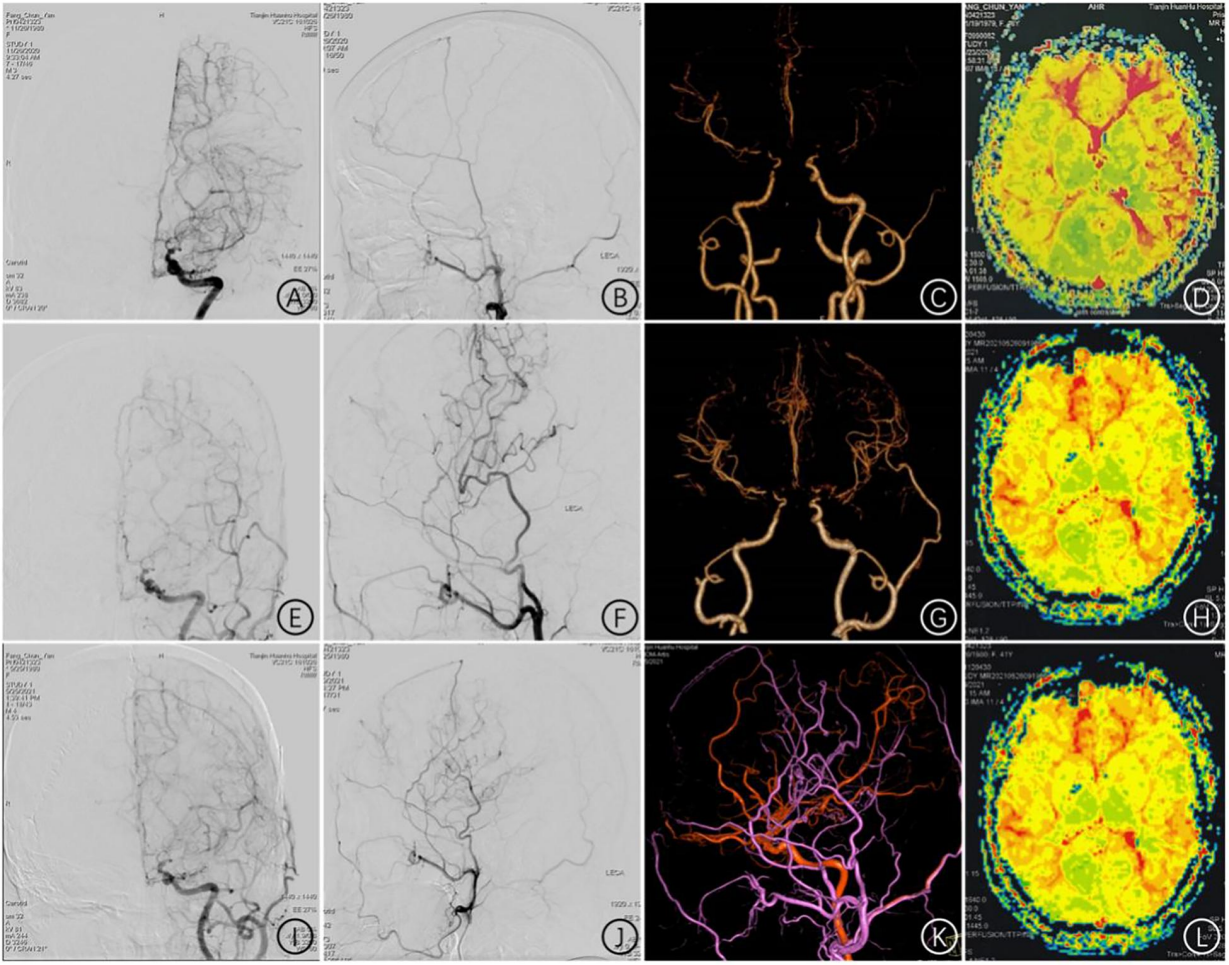
Imaging follow-up of cases of STA-MCA + pedicled temporoparietal fascia application combined with blood flow reconstruction surgery **(A)** preoperative DSAT indicated stenosis at the end of the left internal carotid artery and smoke vessel formation; **(B)** preoperative DSA lateral angiography of the left external carotid artery; **(C)** preoperative CTA showed stenosis at the ends of bilateral internal carotid arteries; **(D)** preoperative PWI indicated hypoperfusion in the left cerebral hemisphere; **(E,F)** angiography of the left external carotid artery 5 days after the operation indicated unobstructed STA-MCA; **(G)** CTA 10 days after the operation indicated that the STA-MCA was unobstructed; **(H)** postoperative PWI indicated an improvement in hypoperfusion of the left cerebral hemisphere; **(I,J,K)** angiography reexamination 6 months after the operation indicated that the left STA-MCA remained unobstructed; **(L)** six months after the operation, PWI indicated improved perfusion in the left cerebral hemisphere.

Among the 48 surgical patients included in the STA-MCA + EMS group, 22 cases underwent double-vessel STA-MCA + EMS and 26 cases underwent single-vessel STA-MCA + EMS, and they were divided into the double-vessel group and the single-vessel group (Supplementary Table S3). In the double-vessel group, 10 cases (45%) of patients had their lesions located on the left side (dominant side), and 12 cases (55%) had them on the right side (Figure 3A); The number of patients with lesions located on the left and right sides in the single-vessel group was 9 (46%) and 17 (64%), respectively (*P* = 0.639) (Figure 3B). There were no significant differences in mRS Scores between the double-vessel group and the single-vessel group before surgery, 1 week after surgery, and 6 months after surgery (*P* > 0.05) (Figures 3C–E). The anastomotic patency rate of bypass vessels in both groups at 6 months after surgery (95% in the double-vessel group and 88% in the single-vessel group) was slightly lower than that at 1 week after surgery (100% in both the double-vessel group and the single-vessel group), but there was no statistically significant difference in the anastomotic patency rate between the groups at 1 week and 6 months after surgery (Figures 3F,G). There was no difference in CBF and MTT values between the double-vessel group and the single-vessel group at the same postoperative follow-up time (Figures 3H–K). In the same group, the reexamination of perfusion indicators (CBF, MTT) at 6 months after the operation was compared with those before the operation and 1 week after the operation. CBF was significantly increased (*P* < 0.05), and MTT was significantly decreased (*P* < 0.05) (Supplementary Table S4, Figure 4). Figures 5, 6 present the follow-up imaging results for the two groups of surgical cases, respectively.

## 4 Discussion

### 4.1 Comparative analysis of STA-MCA + TPFF and STA-MCA + EMS in the treatment of Moyamoya disease

This group of studies retrospectively compared and analyzed patients with moyamoya disease treated by STA-MCA + TPFF and STA-MCA + EMS. The research findings indicated that the incidence of postoperative epilepsy in the TPFF group was significantly lower than that observed in the EMS group (*P* = 0.043). The author posits that this difference may be attributed to the fact that, following TPFF application on the brain surface, there is an absence of potential intracranial compression, which prevents any significant space-occupying effects. Additionally, this approach can mitigate electromyographic activity induced by temporal muscle manipulation, thereby reducing the risk of postoperative epilepsy (4). The results of this study also revealed that there were no significant differences between the two groups in postoperative complications (such as cerebral hemorrhage, cerebral infarction, poor wound healing, transient neurological dysfunction, etc. one week after surgery), mRS Scores (one week and six months after surgery), postoperative bypass patency rate (one week and six months after surgery), and Matsushima classification during postoperative follow-up. Therefore, this study posits that the STA-MCA + TPFF procedure and the STA-MCA + EMS procedure demonstrate comparable clinical efficacy and prognosis in the treatment of Moyamoya disease. Furthermore, the bypass patency rate was observed to be 100% one week post-operation in both groups; however, a slight decrease was noted at six months following the surgery. (94% in the EMS group and 97% in the TPFF group).The author posits that this phenomenon may be attributed to several factors, including postoperative vascular spasm, intimal hyperplasia of blood vessels, alterations in blood flow shear stress, and the development of collateral circulation (3, 7, 8).

Based on the exploration of anatomical structures and clinical practices in the early stages of this study, along with the findings from this research and previous scholarly achievements, this group of studies posits that the STA-MCA + TPFF surgical method offers several advantages: (1) The TPFF exhibits a relatively large fascia area, measuring approximately 6 cm by 8 cm. It is characterized by dual systems of arteries and veins, along with an abundant capillary network. (2) The TPFF has the capability to cover the surface of the cerebral cortex, while simultaneously allowing direct contact with the pia mater. This characteristic is particularly advantageous for promoting the formation of new blood vessels; (3) After the application of TPFF to the brain surface, there is no risk of intracranial compression, and significant space-occupying effects are unlikely to occur. Additionally, this approach mitigates the potential for postoperative epilepsy that may be induced by myoelectrical activity following the use of temporal muscle; (4) TPFF is thinner than the temporal muscle, which allows for improved cosmetic outcomes. This characteristic maximizes the preservation of the patient's appearance, reduces potential damage to the temporal muscle, and minimizes unnecessary intracranial space-occupying effects. Additionally, it helps avoid adverse impacts on the masticatory function of the patient's temporal muscle resulting from any associated damage. (5) TPFF features an arteriovenous dual system that effectively prevents the filling and swelling of the flap vein. This mechanism ensures adequate inflow and outflow of the superficial temporal artery (STA) while mitigating adverse reactions such as postoperative hyperperfusion syndrome or hemodynamic disorders, which may arise from a rapid increase in intracranial blood flow following direct blood flow reconstruction between the STA and middle cerebral artery (MCA). Consequently, TPFF presents several potential advantages over EMS.

### 4.2 Comparative analysis of single and double STA-MCA + EMS in the treatment of Moyamoya disease

Although numerous studies have demonstrated that direct blood flow reconstruction of the superficial temporal artery to middle cerebral artery (STA-MCA) effectively treats Moyamoya disease by enhancing cerebrovascular blood supply and reducing the risk of postoperative stroke, the incorporation of indirect blood flow reconstruction may also facilitate the formation of additional collateral circulation in the long term (9, 10). However, following traditional single-vessel STA-MCA bypass surgery, some patients continue to experience inadequate improvement in their postoperative cerebral ischemia symptoms or may develop complications such as postoperative cerebral hyperperfusion syndrome and transient neurological dysfunction (11, 12). Currently, some studies indicate that the treatment of Moyamoya disease using double-vessel STA-MCA bypass is both feasible and effective. This scholar posits that by enhancing direct blood flow through the double-vessel STA and redistributing blood flow to various ischemic regions of the MCA, a high rate of anastomotic patency can be achieved (13, 14). However, in clinical practice, the double-vessel STA-MCA technique has not been extensively utilized for the treatment of moyamoya disease. This may be attributed to factors such as prolonged operation times and heightened technical demands associated with this surgical approach. The specific application value of this method remains to be explored further (15). Based on this, it is essential for this study to analyze the differences in postoperative efficacy, safety, and complications between the two surgical techniques: single STA-MCA + EMS and double STA-MCA + EMS. This analysis aims to explore the feasibility and application value of double STA-MCA surgery in treating Moyamoya disease, thereby providing a reference framework for its surgical management.

Previous studies have suggested that (15), Compared to the single-vessel group, the double-vessel group demonstrated greater efficacy in treating ischemic cerebrovascular diseases and provided a more substantial replenishment of blood flow. However, this body of studies revealed no significant differences in mRS scores between the two groups. (before surgery, 1 week after surgery, and 6 months after surgery), postoperative anastomotic patency rates of bypass vessels (1 week after surgery and 6 months after surgery), and Matsushima grades between the double-vessel group and the single-vessel group (*P* > 0.05). This finding is inconsistent with previous studies. The results of the current study did not demonstrate this advantage. Furthermore, these results indicate that both single-vessel and double-vessel bypass procedures can be utilized to treat patients with Moyamoya disease; however, single-vessel bypass appears to achieve superior clinical efficacy. Additionally, this group of studies found that the bypass patency rate was 100% in both groups one week post-operation; however, it decreased in both groups six months after the procedure. (95% in the double-vessel group and 88% in the single-vessel group).This is consistent with the research results of Peter et al. (13) (The vascular patency rate in the double-vessel ST-MCA bypass group was 100%, and the patency rate at long-term follow-up was 98.1%).

In this study, CTP perfusion imaging indicators (CBF, MTT) were utilized to assess the characteristics of blood perfusion changes in the surgical area between the two groups at three time points: before surgery, one week post-surgery, and six months post-surgery. The results indicated that perfusion indicators for both groups improved within one week after surgery. By six months post-surgery, these indicators were significantly better than those recorded before surgery and one week after surgery; however, no significant difference was observed between the two groups. This may be attributed to the fact that most patients with moyamoya disease possess a potential collateral circulation network, allowing a single donor vessel to provide adequate blood flow through collateral pathways.

A recent study conducted a retrospective analysis of 28 patients diagnosed with moyamoya disease who underwent STA treatment. The findings indicated no significant difference in the incidence of perioperative complications between double-vessel bypass and single-vessel bypass techniques (16). Some scholars contend that, in comparison to single-vessel bypass surgery, double-vessel bypass surgery has the potential to enhance blood supply and effectively direct blood flow to various ischemic regions of the middle cerebral artery (MCA) within the frontal and temporal lobes. Importantly, this approach does not appear to elevate the risks of epilepsy, hyperperfusion, bleeding, or cerebral infarction (17–19). A total of 22 cases underwent double-bypass grafting in this study. The follow-up results indicated that there was no significant difference in postoperative symptom improvement or complication rates between the double-bypass group and the single-bypass group. This finding not only aligns with previous studies but also further substantiates that both the single-bypass and double-bypass approaches are effective and safe for treating Moyamoya disease.

The research data presented in this article were obtained from the neurosurgery departments of two distinct hospitals. To ensure the consistency of the study, all surgical procedures were meticulously conducted in accordance with the methodologies outlined in the methods section of this article. However, it is unavoidable that different surgeons may approach certain details differently. For example, individual surgeons often exhibit unique personal styles regarding micro-techniques such as arterial separation methods and vascular protection strategies. Additionally, variations in postoperative management practices can also significantly influence the incidence of complications. Furthermore, there is a possibility that some patients' symptoms may be overlooked due to delays in observation. In the future, we aim to further increase the sample size to minimize errors arising from regional variations. Concurrently, the exploration of novel surgical techniques represents a significant avenue for advancing the treatment of Moyamoya disease.

Based on the experiences summarized from laboratory anatomy and clinical surgical practices during the initial phase of this study, along with the findings of this research and previous scholarly contributions, this group of studies posits that double-vessel ST-MCA blood flow reconstruction offers several advantages.: (1) Dual-channel STA blood flow can be distributed to a broader area or various lobe regions without depending on the anterograde and retrograde redistribution of anastomotic blood flow; (2) It has the potential to facilitate a greater flow of bypass, thereby enhancing cerebral blood perfusion. This approach may be particularly advantageous for patients with compromised vascular conditions or inadequate collateral circulation; (3) Double-vessel bypass can ensure that the alternative vessel serves as a backup for subsequent blood perfusion in the event of occlusion of one vessel. Additionally, no extra surgical incisions are necessary. However, it is important to note that double-vessel STA-MCA blood flow reconstruction also presents certain limitations; (1) Two anastomoses are necessary, which complicates the surgical procedure and extends the duration of the operation; (2) If the frontal or apical branch of the superficial temporal artery (STA) is excessively thin, this surgical procedure may not be suitable, and the postoperative patency rate is likely to be low; (3) The occurrence of postoperative cerebral hyperperfusion syndrome highlights the necessity for individualized and judicious selection of surgical methods. It is essential to maximize the advantages of the double-vessel bypass technique while ensuring the safety of the procedure, which warrants further investigation.

## 5 Summary

This study presents a comparative analysis of the STA-MCA + TPFF surgical approach in relation to the traditional STA-MCA + EMS technique, aiming to further investigate the feasibility and effectiveness of the STA-MCA + TPFF method.

The STA-MCA + TPFF combined blood flow reconstruction technique is both safe and effective for the treatment of adult Moyamoya disease. Its clinical efficacy, postoperative complications, prognosis, and other relevant aspects are largely comparable to those associated with the STA-MCA + EMS surgical method. Furthermore, this approach offers several advantages, including reduced trauma, a simpler operative procedure, and the avoidance of adverse reactions linked to temporal muscle application. Therefore, it holds promise for clinical promotion and application.
Both double-vessel STA-MCA + EMS and single-vessel STA-MCA + EMS techniques have been shown to effectively enhance cerebral blood perfusion in patients with moyamoya disease (MMD). There are no significant differences between the two approaches regarding clinical efficacy, postoperative complications, prognosis, and other relevant factors. The double-vessel STA bypass may serve as a complementary option to the single-vessel STA bypass surgical method.

## Data Availability

The original contributions presented in the study are included in the article/Supplementary Material, further inquiries can be directed to the corresponding authors.
