# Correction: Research on the application of cerebral blood flow reconstruction technology in the surgical treatment of moyamoya disease

**DOI:** 10.3389/fsurg.2026.1800200

**Published:** 2026-02-20

**Authors:** Yuyang Chen, Chaojue Huang, Song Wu, Chang Liu, Yu Luo, Litian Huang, Guan Cao, Hui Liang, Panlin Mo, Jiachao Lu, Xiangsheng Su, Xiaoguang Tong, Daqin Feng, Tang Li

**Affiliations:** 1 Department of Neurosurgery, Wuming Hospital Affiliated to Guangxi Medical University, Nanning, China; 2 Department of Neurosurgery, The First Affiliated Hospital, Guangxi Medical University, Nanning, China; 3 Department of Neurosurgery, Tianjin Huanhu Hospital, Tianjin, China

**Keywords:** combined blood flow reconstruction, middle cerebral artery, moyamoya disease, superficial temporal artery, temporoparietal fascia flap

Authors Chaojue Huang, Song Wu, Chang Liu were erroneously omitted as equal contributing authors and co-first authors.

The original version of this article has been updated.

