# Supplementary material for: Research on the application of cerebral blood flow reconstruction technology in the surgical treatment of moyamoya disease
Source: Front Surg. 2026 Jan 26;13:1726401. doi: 10.3389/fsurg.2026.1726401 (PMC12883822; doi:10.3389/fsurg.2026.1726401)
Supplement: Supplementary file 1 [file Supplementaryfile1.docx]

Surgical methods

（1）Single vessel (Supplementary Figure 1)/double vessel (Supplementary Figure 2) STA-MCA combined with EMS surgical procedures

①Based on the preoperative imaging of the patient and touching the superficial temporal artery, the course of the target blood vessel was marked in the projected area on the patient's body surface, and the incision was designed according to the selected target blood vessel.

②Cut open the scalp, expose the superficial temporal artery, and expose the frontal branch and/or parietal branch for bypass. Expand the scalp incision. Cut the temporal muscle to form a myofascial flap.

③Bone flap craniotomy is performed to form a bone window. Hemostasis of the wound surface and suspension of the dura mater.

④Cut the dura mater and arachnoid membrane in an arc sequence, and release the cerebrospinal fluid slowly and appropriately to reduce the tension of the brain tissue.

⑤Continue to separate the frontal and/or parietal branches of the superficial temporal artery. After good exposure, cut the distal end and insert it into the cranium through the temporal muscle.

⑥Carefully separate and fully expose the branches of the middle cerebral artery on the surface of the frontotemporal lobe.

⑦During the operation, fluorescence angiography was performed in combination with Flow800 imaging (for specific steps, please refer to 1.5, Figure 4). The appropriate recipient vessels were selected, and the frontal branch and/or parietal branch were anastomoted with the ends of the M4 segment of the middle cerebral artery near the lateral fissure.

⑧After the intracranial blood vessels were successfully reconstructed, hemostatic materials were used to stop the bleeding.

⑨After the completion of intracranial and extracranial bypass grafting, fluorescence angiography confirmed that the anastomosis was unobstructed, the donor and recipient vessels were unobstructed, and the compensatory blood supply range was good.

⑩The wound was thoroughly hemostatic, the dura mater was tightly sutured, the skull was repositioned and fixed, the temporal muscle fascia flap and flap were repositioned, the incision was sutured in layers, and pressure bandaging was applied. The operation was completed.

（2）Surgical procedures for STA-MCA bypass combined with TPFF application（Supplementary Figure 3）：

①Based on the preoperative imaging of the patient (including clarifying the direction of the head and facial skin to the branches of the superficial temporal artery, especially the frontal and parietal branches, under the guidance of vascular ultrasound before the operation) and touching the superficial temporal artery, the projected area of the target vessel's course on the patient's body surface was marked, and the incision was designed according to the selected target vessel.

②An arc-shaped incision about 8cm long above the zygomatic arch in front of the frontotemporal ear was designed along the course of the superficial temporal artery, and the scalp was incised to expose the superficial temporal artery and separate the main trunk and parietal branch of the superficial temporal artery.

③Starting from the superior medial periosteum, gradually transitioning to the temporoparietal fascia and then extending downward to the lateral side, a TPF of approximately 6 cm×8 cm was stripped from the surface layer of the deep temporal fascia. The flap was excised while retaining a pedicle in the direction of the superficial temporal artery and facilitating rotation.

④Push the flap from the temporal region towards the zygomatic arch area. During the operation, it is necessary to protect the branches of the facial nerve and keep the surgical area moist with normal saline gauze. Cut the distal end of the top branch of the superficial temporal artery, rinse the lumen with heparinized papaverine saline, and temporarily block the distal end for later use.

⑤Continue to cut the temporal muscle, drill a hole in the skull, and milled the skull with a milling cutter to form a 4*6cm bone window. Suspend the dura mater and stop bleeding from the wound.

⑥Make an arc-shaped incision of the dura mater, and pay attention to protecting the main branches of the middle meningeal artery.Separate the M4 branch of the middle cerebral artery with relatively thick diameters in the frontal and temporal lobes within the bone window, and cut the arachnoid membrane. The ends of the branches of the superficial temporal artery were dissected approximately 1cm, and the superficial temporal artery - M4 segment of the middle cerebral artery end-to-end anastomosis was performed respectively

⑦During the operation, fluorescence angiography was performed and the appropriate recipient vessels were selected in combination with the results of Flow800 imaging. The frontal branch and/or parietal branch were anastomosed laterally with the M4 segment of the middle cerebral artery near the lateral fissure.

⑧After the successful reconstruction of intracranial blood vessels, the intraoperative blocking time was approximately 15 minutes. Stop the bleeding with hemostatic materials.

⑨After the completion of intracranial and extracranial bypass grafting, fluorescence angiography and Flow 800 confirmed that the anastomosis was unobstructed, the donor and recipient vessels were unobstructed, and the compensatory blood supply range was good.

⑩The wound was thoroughly hemostatic, the dura mater was tightly sutured, the skull was repositioned and fixed, the temporal muscle fascia flap and flap were repositioned, the incision was sutured in layers, and pressure bandaging was applied. The operation was completed.

Flow800-ICGV angiography

①Before or after blood flow reconstruction, ICG is rapidly injected into the selected vein. The dosage of ICG is 0.2mg/kg. Note that the time of the two consecutive injections should be consistent with that of the vein. At the same time, pay attention to recording the patient's blood pressure and its changes during the ICG injection process.

②The target blood vessels were observed in the fluorescence microscopy mode of the Zeiss KINEVO 900 robotic surgical microscope, and the fluorescence intensity-time graph was generated using the Flow 800 software (Supplementary Figure 4).

③Under the fluorescence contrast image, select the surgical field that needs to be observed as the region of interest.

④Analyze the fluorescence intension-time curves of each selected region of interest, and use the FLOW 800 software to automatically calculate the peak fluorescence intensity of the target blood vessel, the time to peak fluorescence intensity, etc. The time to peak fluorescence intensity refers to the period from intravenous injection of ICG to the appearance of peak fluorescence intensity in the region of interest.

⑤Calculate the difference in the peak time of vascular fluorescence intensity in different regions of interest.

**Supplementary Figure 1 The surgical process of single STA-MCA combined with temporal muscle patch for blood flow reconstruction** (A)Refine the incision to a question mark shape positioned anterior to the tragus；(B) separate the main trunk and branches of the superficial temporal artery；(C) separate the temporal muscle to form the frontal temporal bone flap；(D, E) ICG fluorescence angiography assists in the selection of M4 segment receptor vessels；(F, G) perform STA-MCA end-to-end anastomosis；(H) ICG fluorescence angiography showed that the bridge vessels were unobstructed；(I) the temporal muscle is attached to the surface of the cortex and sutured to the dural margin.

**Supplementary Figure 2 The surgical process of double STA-MCA+ temporal muscle application combined with blood flow reconstruction** (A) the frontal and temporal cortex is exposed, with lateral fissures being the most severe；(B) ICG fluorescence angiography assists in the selection of MCA receptor vessels；(C) further vascular selection of the Flow 800 helper receptor was performed；(D)block the MCA；(E) complete the first anastomotic suture；(F） complete the second anastomotic suture；（G）ICG fluorescence angiography was used to monitor the patency of the anastomosis；（H）Flow 800 is used to monitor the patency of the anastomosis and hemodynamic changes（Note: The white arrow points to the STA-MCA anastomosis）

**Supplementary Figure 3 The surgical process of MCA combined with pedicled temporoparietal fascia flap application for blood flow reconstruction** （A） the surgical incision adopts an inverted L-shaped incision in front of the tragus; （B） separate the petted temporoparietal fascia flap；（C） formation of the frontal temporal bone flap；（D） select the appropriate M4 receptor vessels and perform STA-MCA end-to-end anastomosis; （E）ICG fluorescence angiography showed that the bridge vessels were unobstructed；（F) the pedunculated temporoparietal fascia is adhered to the surface of the cortex, and the periphery is sutured to the dural margin。

**Supplementary Figure 4 Selection of Flow 800 helper receptor vessels and comparison before and after end-side anastomosis of STA-MCA** (A) Flow 800 imaging before anastomosis assisted receptor vascular selection；(B) Flow 800 imaging was used to evaluate the patency of the anastomosis and hemodynamic changes after anastomosis.
