# Supplementary material for: Research on the application of cerebral blood flow reconstruction technology in the surgical treatment of moyamoya disease
Source: Front Surg. 2026 Jan 26;13:1726401. doi: 10.3389/fsurg.2026.1726401 (PMC12883822; doi:10.3389/fsurg.2026.1726401)
Supplement: Supplementary file 2 [file Table1.docx]

Table 1 General clinical data of patients in the EMS group and the TPFF group

| Variable | EMS group （n = 48） | TPFF group（n = 31） | *P* |
| --- | --- | --- | --- |
| Gender |  |  | 0.196 |
| Male（case%） | 27 （56%） | 12 （39%） |  |
| female（case%） | 21 （44%） | 19 （61%） |  |
| Age | 42 （29.75, 51） | 39 （30, 52） | 0.98 |
| Family history（case%） | 0 | 0 | 1 |
| First clinical symptoms |  |  | 0.185 |
| hemorrhage（case%） | 19（40%） | 7 （23%） |  |
| Ischemia（case%） | 29 （60%） | 24 （77%） |  |
| Preoperative clinical manifestations |  |  |  |
| Fatigue（case%） |  |  | 1 |
| Yes | 33（69%） | 21 （68%） |  |
| No | 15 （31%） | 10 （32%） |  |
| Aphasia（case%） |  |  | 0.722 |
| Yes | 20 （42%） | 15（48%） |  |
| No | 28（58%） | 16 （52%） |  |
| Blurred vision（case%） |  |  | 1 |
| Yes | 7（15%） | 4（13%） |  |
| No | 41（85%） | 27（87%） |  |
| Dizziness（case%） |  |  | 0.732 |
| Yes | 7（15%） | 3（10%） |  |
| No | 41（85%） | 28（90%） |  |
| Epilepsy（case%） |  |  | 1 |
| Yes | 3（6%） | 1（3%） |  |
| No | 45（94%） | 30（97%） |  |
| Memory decline（case%） |  |  | 0.643 |
| Yes | 4（8%） | 1（3%） |  |
| No | 44（92%） | 30 （97%） |  |
| Headache（case%） |  |  | 0.236 |
| Yes | 6（12%） | 1（3%） |  |
| No | 42（88%） | 30（97%） |  |
| Asymptomatic（case%） |  |  | 1 |

Table 1 (Continued)General clinical data of patients in the EMS group and the TPFF group

| Variable | EMS group （n = 48） | TPFF group（n = 31） | *P* |
| --- | --- | --- | --- |
| Yes | 4（8%） | 2（6%） |  |
| No | 44（92%） | 29（94%） |  |
| Basic diseases |  |  |  |
| Hypertension（case%） |  |  | 0.73 |
| Yes | 11（23%） | 9（29%） |  |
| No | 37（77%） | 22（71%） |  |
| Diabetes（case%） |  |  | 1 |
| Yes | 3（6%） | 2（6%） |  |
| No | 45（94%） | 29（94%） |  |
| Hyperlipidemia（case%） |  |  | 1 |
| Yes | 16（33%） | 10（32%） |  |
| No | 32（67%） | 21（68%） |  |
| Preoperative mRS Score |  |  | 0.325 |
| 0-2（case%） | 43（90%） | 25（81%） |  |
| 3-4（case%） | 5（10%） | 6（19%） |  |
| 5-6（case%） | 0 | 0 |  |
| Suzuki stage（case%） |  |  | 0.937 |
| Ⅰ | 0 | 0 |  |
| Ⅱ | 4（8%） | 2（6%） |  |
| Ⅲ | 29（60%） | 18（58%） |  |
| Ⅳ | 15（31%） | 11（35%） |  |
| Ⅴ-Ⅵ | 0 | 0 |  |

Table 2 Comparison of postoperative observational indicators between the EMS group and the TPFF group

| Variable | EMS （n = 48） | TPFF（n = 31） | *P* |
| --- | --- | --- | --- |
| Surgical side profile |  |  | 0.098 |
| Left（case%） | 19（40%） | 19（61%） |  |
| Right（case%） | 29（60%） | 12（39%） |  |
| Follow-up period (months) |  |  | 0.873 |
| 6 | 37（77%） | 27（87%） |  |
| 7 | 5（10%） | 2（6%） |  |
| 8 | 4 （8%） | 1（3%） |  |
| 9 | 2 （4%） | 1（3%） |  |
| Postoperative complications |  |  |  |
| Cerebral hemorrhage（case%） |  |  | 0.698 |
| Yes | 5（10%） | 2 （6%） |  |
| No | 43（90%） | 29（94%） |  |
| Cerebral infarction（case%） |  |  | 0.112 |
| Yes | 10（21%） | 2（6%） |  |
| No | 38（79%） | 29（94%） |  |
| Epilepsy（case%） |  |  | 0.043 |
| Yes | 10（21%） | 1（3%） |  |
| No | 38（79%） | 30（97%） |  |
| Poor wound healing（case%） |  |  | 0.698 |
| Yes | 5（10%） | 2（6%） |  |
| No | 43（90%） | 29（94%） |  |
| Transient neurological dysfunction（case%） |  |  | 0.744 |
| Yes | 6（12%） | 5（16%） |  |
| No | 42（88%） | 26（84%） |  |
| The clinical manifestations changed one week after the operation |  |  | 1 |
| No change（case%） | 4（8%） | 2（6%） |  |
| Improve/Disappear（case%） | 44（92%） | 29（94%） |  |
| Deteriora（case%） |  |  |  |
| mRS Score one week after the operation |  |  | 0.685 |
| 0-2（case%） | 39（81%） | 26（84%） |  |
| 3-4（case%） | 9（19%） | 5（16%） |  |
| 5-6（case%） | 0 | 0 |  |

Table 2 (Continued) Comparison of postoperative observational indicators between the EMS group and the TPFF group

| Variable | EMS （n = 48） | TPFF（n = 31） | *P* |
| --- | --- | --- | --- |
| Last mRS Score |  |  | 0.835 |
| 0-2（case%） | 45（98%） | 30（98%） |  |
| 3-4（case%） | 3（2%） | 1（3%） |  |
| 5-6（case%） | 0 | 0 |  |
| The vascular patency rate one week after the operation（case%） | 48（100%） | 31（100%） | 1 |
| The vascular patency rate 6 months after the operation（case%） | 45（94%） | 30（97%） | 1 |
| Matsushima Classification |  |  | 0.761 |
| A | 10（21%） | 5（16%） |  |
| B | 27（56%） | 20（65%） |  |
| C | 11（23%） | 6（19%） |  |

Table 3 Comparison of postoperative observational indicators between the double-vessel group and the single-vessel group

| Variable | double-vessel（n=22） | single-vessel（n=26） | t/χ^2^/u | *P* |
| --- | --- | --- | --- | --- |
| Surgical side profile |  |  | 0 | 0.639 |
| Left（case%） | 10（45%） | 9（46%） |  |  |
| Right（case%） | 12（55%） | 17（64%） |  |  |
| The patency rate of the anastomosis one week after the operation（case%） | 22（100%） | 26（100%） | / | 1 |
| The patency rate of the anastomosis 6 months after the operation（case%） | 21（95%） | 23（88%） | 0.145 | 0.34 |
| Preoperative mRS Score |  |  | Fisher | 1.00 |
| 0-2（case%） | 20（91%） | 23（88%） |  |  |
| 3-4（case%） | 2（9%） | 3（12%） |  |  |
| 5-6（case%） | 0（0%） | 0（0%） |  |  |
| mRS Score one week after the operation |  |  | Fisher | 0.151 |
| 0-2（case%） | 20（91%） | 19（73%） |  |  |
| 3-4（case%） | 2（9%） | 7（27%） |  |  |
| 5-6（case%） | 0（0%） | 0（0%） |  |  |
| mRS Score at 6 months after the operation |  |  | Fisher | 1.00 |
| 0-2（case%） | 21（95%） | 24（92%） |  |  |
| 3-4（case%） | 1（5%） | 2（8%） |  |  |
| 5-6（case%） | 0（0%） | 0（0%） |  |  |
| CTP one week after surgery |  |  |  |  |
| CBF（ml/100ml/min） | 50.17±15.72 | 47.82±19.96 | -0.447 | 0.657 |
| MTT（s） | 5.34±1.57 | 6.43±3.74 | 1.268 | 0.211 |
| CTP six months after surgery |  |  |  |  |
| CBF（ml/100ml/min） | 60.52±15.04 | 56.07±15.08 | 1.346 | 0.187 |
| MTT（s） | 4.49±1.25 | 6.43±3.74 | -0.259 | 0.797 |

Abbreviation：t, t value；χ^2^，hi-square value；u，uvalue；CBF，cerebral blood flow；MTT，mean Time Through.

Table 4 The changes of CTP after surgery in the double-vessel group and the single-vessel group

| Indicator | Before surgery | One week after the operation | Six months after the operation | F | *P* value |
| --- | --- | --- | --- | --- | --- |
| CBF |  |  |  |  |  |
| Double-vessel | 50.20±14.82* | 54.19±16.13# | 60.53±15.13# | 8.689 | 0.001 |
| Single-vessel | 47.74±19.87* | 48.19±18.49# | 56.12±14.79# | 5.06 | 0.01 |
| MTT |  |  |  |  |  |
| Double-vessel | 5.42±1.43* | 5.21 ±1.26ns | 4.48±1.19# | 6.314 | 0.004 |
| Single-vessel | 6.43±3.74* | 6.12 ±2.98# | 4.37±1.57# | 7.905 | 0.005 |

Note: * and # indicate that pairwise comparisons are statistically significant (P<0.05), that is, the CBF/ MTT values at 1 week and 6 months after the operation are different from those before the operation.CBF（ml/100ml/min）
