# Supplementary figures and images for: Research on the application of cerebral blood flow reconstruction technology in the surgical treatment of moyamoya disease
Source: Front Surg. 2026 Jan 26;13:1726401. doi: 10.3389/fsurg.2026.1726401 (PMC12883822; doi:10.3389/fsurg.2026.1726401)

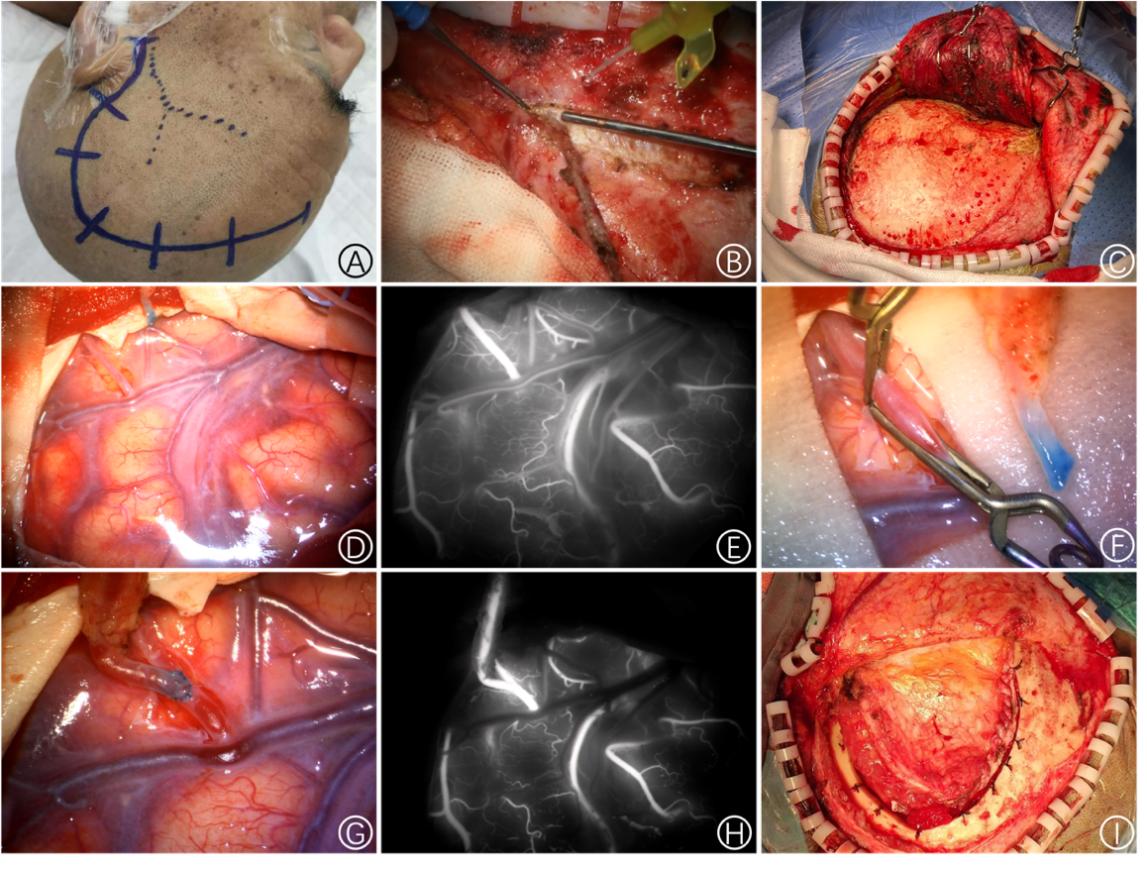

Supplement: Supplementary file 3 [file Image1.jpeg]

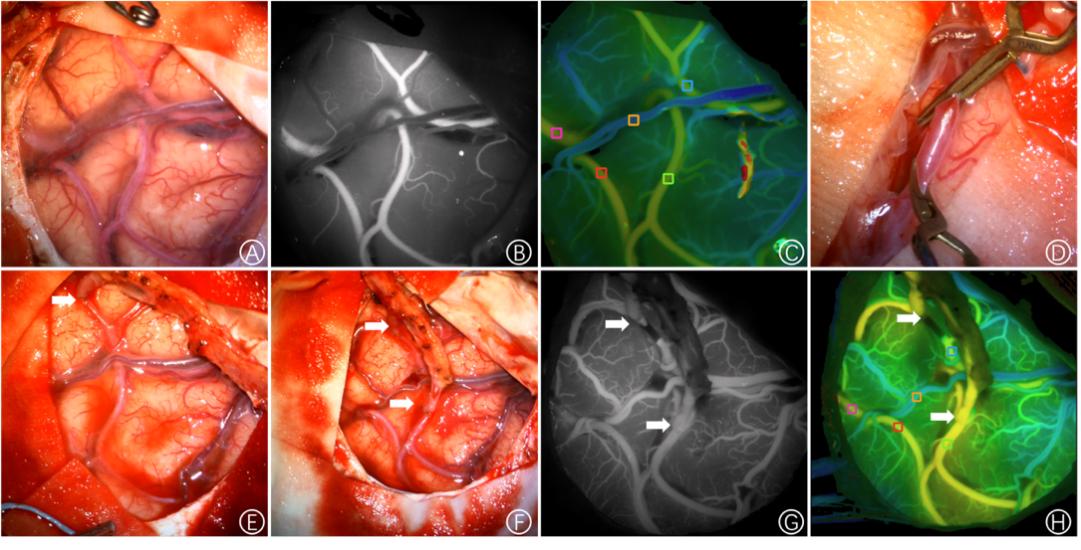

Supplement: Supplementary file 4 [file Image2.jpeg]

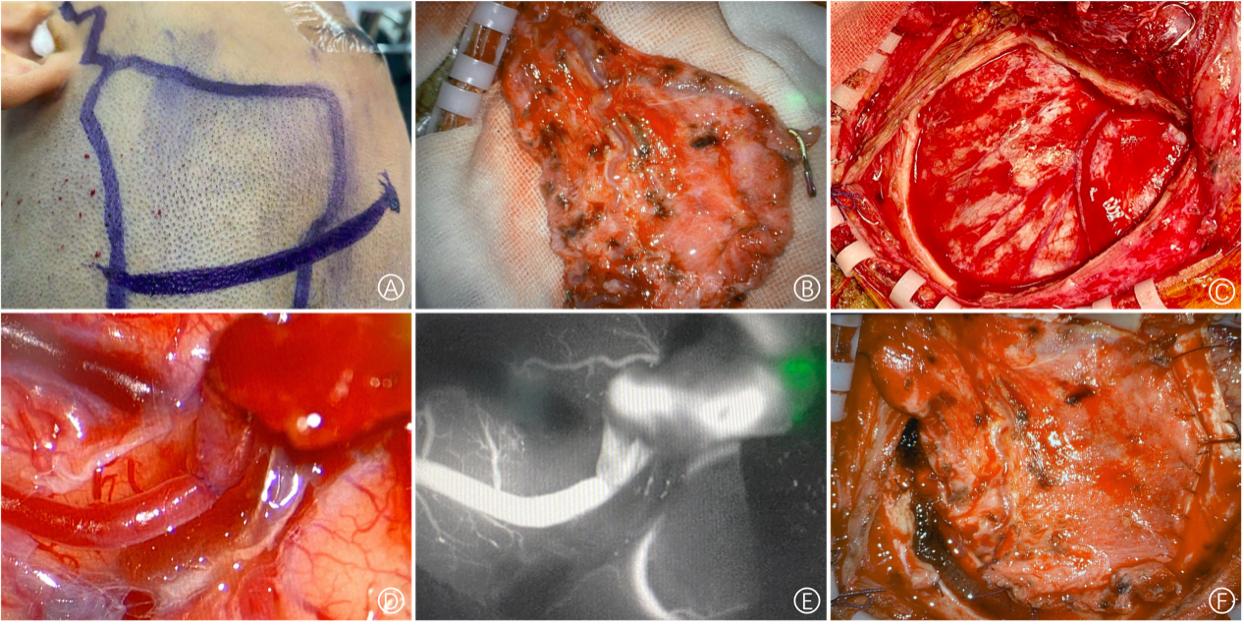

Supplement: Supplementary file 5 [file Image3.jpeg]

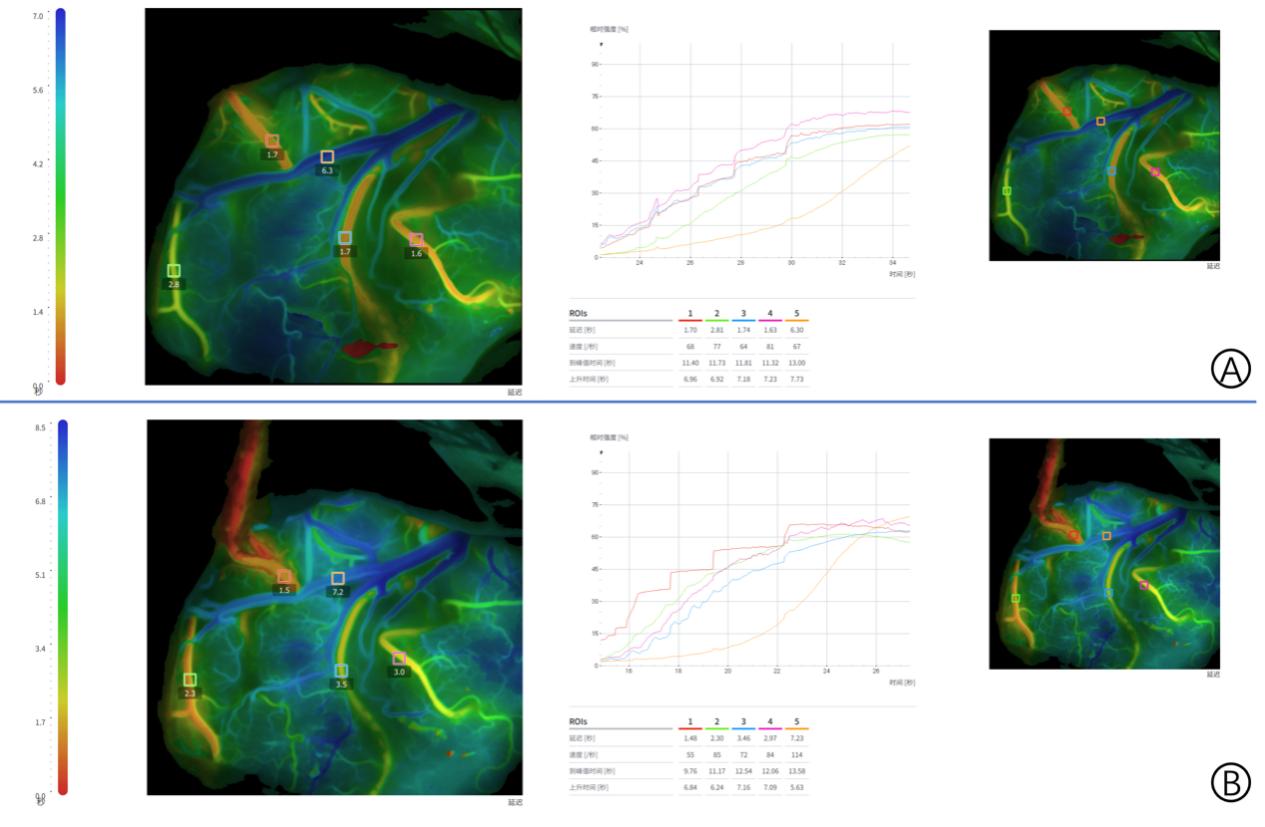

Supplement: Supplementary file 6 [file Image4.jpeg]
